# Supplementary material for: Independent theta phase coding accounts for CA1 population sequences and enables flexible remapping
Source: eLife. 2015 Feb 2;4:e03542. doi: 10.7554/eLife.03542 (PMC4383210; doi:10.7554/eLife.03542)
Supplement: Supplementary file 1. — Mathematical appendices. This file includes all mathematical methods and derivations pertaining to the models described in the main text. DOI: http://dx.doi.org/10.7554/eLife.03542.022 [file elife03542s001.pdf]

Mathematical Appendices

A1: Single Cell Phase Coding Model

Here, we provide a simple mathematical description of the phase and frequency of a place cell as a function of the animal's location, running speed and the place field size. The properties of the cell's firing rate are discussed in the following section.

Consider a place field with center x_c on a linear track. We define the encoded phase $\phi(x)$ which assigns a firing phase to each location x inside the place field. The parameters describing the encoded phase are the firing phase at place field entry ϕ_0, the total phase precessed $\Delta\phi$, and the distance over which this phase is precessed $2R$. The encoded phase is given by:

$$\phi(x) = \phi_0 - \Delta\phi \frac{x - x_c + R}{2R} \tag{A1.1}$$

so that the phase at place field entry $x_c - R$ is ϕ_0 and at place field exit $x_c + R$ is $\phi_0 - \Delta\phi$ as required. The parameter R therefore determines the spatial range of phase precession, whereas the width of the firing rate field σ is considered separately in the following section.

If the rat moves at a constant running speed v, starting from a position x_s at time $t = 0$, and the LFP theta phase has a constant frequency f_θ with an initial phase offset θ_s, then:

$$x(t) = x_s + vt \tag{A1.2}$$
$$\theta(t) = \theta_s + 2\pi f_\theta t \tag{A1.3}$$
$$\phi(t) = \phi_0 - 2\pi f_\phi (t - t_0) \tag{A1.4}$$

where $t_0 = (x_0 - x_s)/v$ is the time that the rat reaches location $x_0 = x_c - R$, the start of the place field. Combined with Equation (A1.1), this gives the rate of phase precession

$$f_\phi = \frac{\Delta\phi}{2\pi} \frac{v}{2R} \tag{A1.5}$$

and provides a complete description of the firing phase of a cell for arbitrary place field locations, running speeds, LFP phase offsets and initial locations on a linear track.

A2: Analysis of Dual Rate and Phase Coding

Equations (1) and (2) in the main text describe the activity of a single cell as a function of the encoded phase $\phi(x)$, LFP theta phase $\theta(t)$ and the animal's location x. In general, these three variables all evolve in time so that we can consider these equations in time only. For a constant running speed (Equations (A1.2, A1.3, A1.4)), the firing rate for a cell during place field crossing is:

$$r(t) = r_x(x(t)) r_\phi(\phi(x(t)) - \theta(t)) \tag{A2.1}$$
$$= A \exp\left(-\frac{(x_s + vt - x_c)^2}{2\sigma^2}\right) \exp\left(k \cos(2\pi(f_\theta + f_\phi)t - \phi_0 + \theta_s - 2\pi f_\phi t_0)\right) \tag{A2.2}$$

Figure 2E shows the firing rate distribution for different values of k. Note that the phase precessed across the firing rate field depends on the relative values of R and σ. Hence, to model ranges of phase precession other than 2π, we are free to fix $\Delta\phi = 2\pi$ and vary R.

The number of spikes fired by a place cell is uncorrelated with the running speed of the animal, and is around $10 - 20$ spikes per pass (Huxter et al., 2003). Hence, using Equation A2.2, the amplitude A was set such that $\int_{-\infty}^{\infty} r(t)dt = N_{\text{spikes}}$, which normalizes the average number of spikes fired per pass through the place field to N_{spikes} (Figure 2-figure supplement 1). A good approximation to this integral can be obtained by setting r_ϕ to be a constant in order to average out its oscillatory component:

$$\langle r_\phi \rangle = \frac{1}{T}\int_0^T r_\phi(t)dt = I_0(k) \tag{A2.3}$$

where $I_0(k)$ is the modified Bessel function of order zero and T is the period of $r_\phi(t)$. Hence, the firing rate of the cell is modulated by running speed as:

$$A(v) \approx \frac{N_{\text{spikes}}}{I_0(k)} \frac{v}{\sqrt{2\pi\sigma^2}} \tag{A2.4}$$

This approximation is accurate when the number of oscillatory cycles within the place field is high. For very high running speeds (or small place fields), this approximation will be less accurate.

Sequence Properties for Independent Coding in the High Phase Locking Limit

Here, we derive analytical expressions for two key quantities which are often measured in experiments: the compression factor and the sequence path length. These expression are strictly only valid in the limit that phase locking is strong, but are useful for understanding how behavioral variables such as running speed can influence sequence properties. In this section, we assume that the activity of each cell is governed by Equation (A2.2), i.e. that the activity of each cell depends only on the animal's location in the place field and the LFP theta phase, and not explicitly on the activity of other cells in the population.

The compression factor measures the ratio between the sequence compressed timescale and the behavioral timescale along a trajectory. Given any two cells active within the same theta cycle, the compression factor is defined as:

$$c = \frac{\Delta t_0}{\Delta t_{\text{spike}}} \tag{A2.5}$$

where Δt_0 is the time it takes the animal to travel between the place field centers of the two cells and Δt_{spike} is the time lag between the spikes of the two cells within the theta cycle (Figure A1). Note that some studies invert this definition of c (e.g., Geisler et al., 2010). These two timescales are generally measured in experiments as peaks in the cross-correlogram of the cell pair.

In the limit of high phase locking ($k \to \infty$), the spikes occur exactly at the peaks of the phasic tuning curve, i.e.:

$$\theta(t_{\text{spike}}) - \phi(t_{\text{spike}}) \equiv 0 \mod 2\pi \tag{A2.6}$$

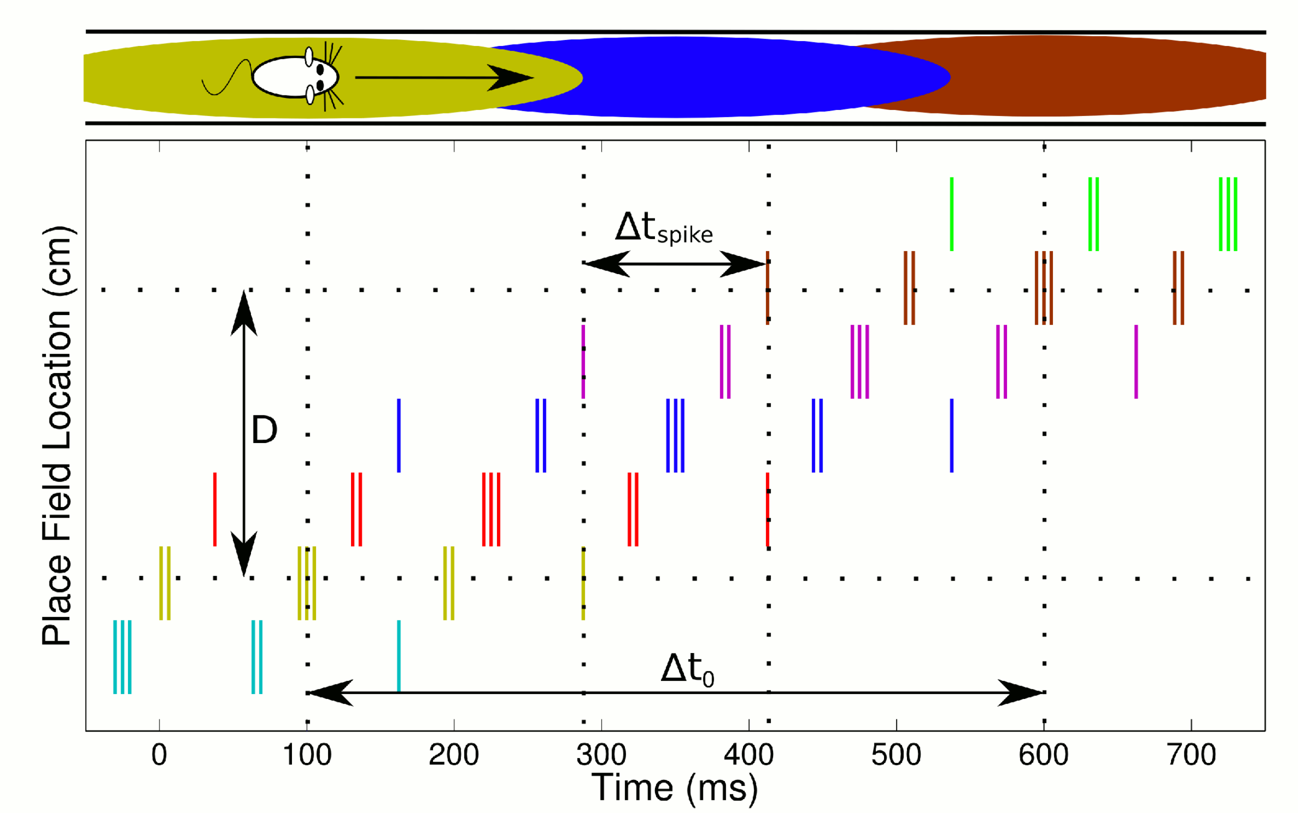

Figure A1: **Definitions of compression factor and sequence path length.**
Shown are the sequence path length D, which is defined as the largest distance between place field centers for any two cells active in a single theta cycle, the time Δt_0 to travel between place field centers of a pair of cells and the time interval Δt_{spike} between spikes fired in a theta cycle by this cell pair. The compression factor is the ratio of these two timescales. (The cell pair illustrated is the first and last cell on the track above, intermediate place fields are omitted on the track.)

Figure A2 provides a graphical illustration of the spike sequence in this limit. Using Equations (A1.3) and (A1.4) in this limit:

$$t_{\text{spike}} = \frac{(\phi_0 - \theta_s) / (2\pi) + f_\phi t_0 + n}{f_\theta + f_\phi} \tag{A2.7}$$

where n is an integer introduced to account for the resetting of phases $\phi(t)$ and $\theta(t)$ each cycle under the modulo arithmetic. If we further assume that each cell precesses over a range of phases of one cycle or less $0 \leq \phi < 2\pi$, then the integer n is simply an index labeling the LFP theta cycle, since ϕ is never reset. Hence, when calculating Δt_{spike} for a cell pair, we can assume n is the same for each spike, since we are considering only spike sequences contained within a single theta cycle. Assuming the cells also share the same phase precession parameters ϕ_0 and f_ϕ, we find $\Delta t_{\text{spike}} = \Delta t_0 f_\phi / (f_\theta + f_\phi)$, so that the compression factor is:

$$c = 1 + \frac{f_\theta}{f_\phi} = 1 + f_\theta \frac{2\pi}{\Delta\phi} \frac{2R}{v} \tag{A2.8}$$

The sequence path length D measures the distance swept out by a sequence during a theta cycle

(Figure A1). We define this as the difference between the maximum and minimum place field locations of cells active in a single theta cycle $D = \max(\Delta x_c) = \max(x_c) - \min(x_c)$. Again, we take the limit $k \to \infty$ where spike times are given by Equation (A2.6). The distance between place field centers is $\Delta x_c = v\Delta t_0 = cv\Delta t_{\text{spike}}$, and the sequence path length D is the largest distance Δx_c for cells with spikes within the time window of one theta cycle, $\Delta t_{\text{spike}} \leq 1/f_\theta$:

$$D = \frac{v}{f_\theta}c = \frac{2\pi}{\Delta\phi}2R + \frac{v}{f_\theta} \tag{A2.9}$$

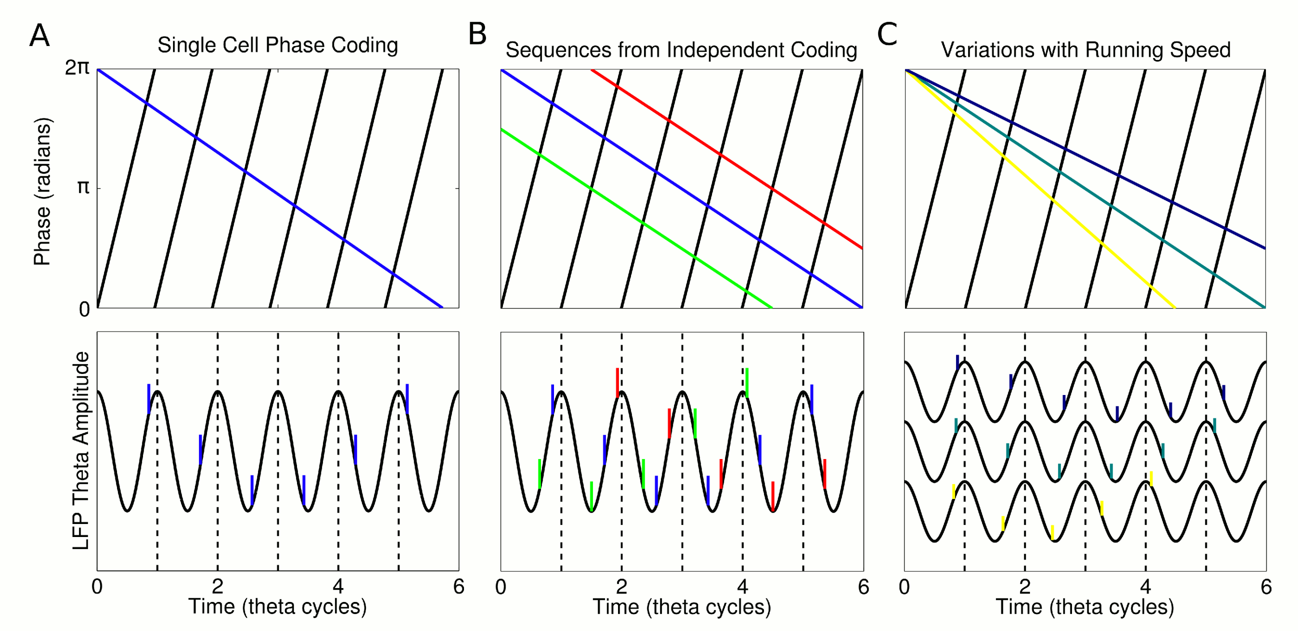

Figure A2: **Sequences in the high phase locking limit ($k \to \infty$).**
Black lines show LFP theta phase $\theta(t)$, colored lines show single cell encoded phase $\phi(t)$. Spikes occur when the encoded phase equals the LFP phase. (A) Single cell coding. (B) A sequence generated through independent phase coding in three cells. (C) Running speed v determines the slope of phase precession f_ϕ in single place cells, which in turn affects sequence properties. Here, three separate runs through the same place field at different speeds are shown for comparison.

Single Cell Coding with Trial to Trial Variability

For completeness, we note here that some studies suggest a more complex single cell coding scheme than that outlined above. In particular, phase precession in single trials may reflect a greater degree of coordination against the theta rhythm than that suggested from the pooled data (Schmidt et al., 2009). The key differences observed in single trials compared to surrogate trials sampled from the pooled data were a higher phase-position correlation and slope and a lower phase range and spatial range. The single cell model we developed above can be extended to incorporate such properties by replacing the fixed phase precession parameters with random variables which vary from trial to trial. For example, a single trial phase offset ϕ_0 sampled from a normal distribution with a variability of $\sigma_{\phi_0} = \pi/2$, together with a fixed phase range of $\Delta\phi = \pi$ on each trial gives a pooled phase range of 2π as well as a lower pooled phase position-correlation, in line with experimental observations.

If, on each trial, each cell samples its parameters from these distributions independently of other cells, the population code will be independent. If instead this trial to trial variability is shared across the population a coordinated population code will result. For simplicity, we will not consider these trial to trial properties of phase precession in the following analyses.

A3: Derivation of Traveling Wave Dynamics for Independent Coding

We now extend the single cell firing rate model to the population behavior under the assumption that each cell is governed independently by the above single cell coding model. Note that, in Equation (A2.2), we have eliminated the rat's location x in favor of time t, since we have assumed a constant running speed. We would like to understand the firing rate in the population both as a function of time t, and place field center x_c. In Equation (A2.2), the remaining t_0 can be expressed in terms of x_c as $t_0 = (x_c - R - x_s)/v$. We define the total phase of each cell as $\psi = \phi - \theta$, which gives :

$$\psi(x_c, t) = 2\pi(f_\theta + f_\phi)t - 2\pi\frac{f_\phi}{v}(x_c - x_s) + \frac{\Delta\phi}{2} - \phi_0 + \theta_s \tag{A3.1}$$

We can set the initial position of the rat as $x_s = 0$ for simplification. Inspecting the structure of this population phase, we see it has the form $\psi(x_c, t) = \omega t - \kappa x_c + \psi_s$. This is the form of a traveling wave of angular frequency $\omega = 2\pi f$, wavenumber $\kappa = 2\pi/\lambda$ and phase offset ψ_s. The frequency and wavelength of the traveling wave are:

$$f(v) = f_\theta + f_\phi(v) \tag{A3.2}$$

$$\lambda = \frac{v}{f_\phi} = \frac{2\pi}{\Delta\phi}2R \tag{A3.3}$$

so that the frequency increases with running speed, but the wavelength stays constant. Hence, the propagation speed of the wave is:

$$v_p = f\lambda = (f_\theta + f_\phi)\frac{v}{f_\phi} = cv \tag{A3.4}$$

and therefore we find an alternative expression for the compression factor derived earlier $c = v_p/v$, i.e. the compression factor is the ratio of the propagation speed of the traveling wave to the speed of the envelope. The propagation speed shows a constant relationship to the running speed of the rat:

$$v_p = v + \frac{2\pi}{\Delta\phi}2Rf_\theta \tag{A3.5}$$

At this point, we also see that (assuming $\Delta\phi = 2\pi$) the sequence path length in Equation (A2.9) is just the distance traveled by this wave in a theta cycle, since $D = v_p/f_\theta$. Putting everything together, the population firing rate is given by:

$$r(x_c, t) = A\exp\left(-\frac{(x_c - vt)^2}{2\sigma^2}\right)\exp\left(k\cos\left(\frac{2\pi f}{cv}(cvt - x_c) + \psi_s\right)\right) \tag{A3.6}$$

which is an equivalent form of Equation (A2.2) and was used to produce Figure 3.

Finally, it is useful to inspect the phase offset ψ_s of the traveling wave. In particular, if we assume a full cycle of phase precession $\Delta\phi = 2\pi$ starting at $\phi_0 = 2\pi$, with initial position $x_s = 0$, we see from Equation (A3.1) that the population phase offset is $\psi_s = \theta_s - \pi$. The maximum population activity occurs at the trough of LFP theta, where zero LFP phase is defined as the peak. Hence, there is a half cycle phase shift between the population waveform and the LFP waveform (although experimentally this effect depends on the recording depth, since the LFP phase varies with depth (Buzsáki, 2002)).

Analysis of Population Theta Rhythm

Here, we show how a slower LFP theta rhythm arises within the population, providing a complementary and fully compatible perspective to that previously given by Geisler et al. (2010). In particular, we see from the above analysis that the frequency of a single cell is higher than the LFP theta since $f = f_\theta + f_\phi$. However, if we change variables to view the traveling wave in coordinates comoving with the envelope, the fast population activity is decoupled from the slow movement of the animal, allowing the population theta frequency to be analyzed.

To see this, we make the change of coordinates $X = x_c - vt$, after which $\psi(X = 0, t)$ measures the phase of the traveling wave relative to the slow moving envelope, since $X = 0$ is the center of the envelope. In these coordinates, the population phase is:

$$\psi(X, t) = \theta(t) - 2\pi \frac{X}{\lambda} \tag{A3.7}$$

This equation shows that $\theta(t) = \psi(X = 0, t)$, and therefore that the LFP theta phase is equal to the phase difference between the traveling wave and envelope. To further illustrate this point, we note that the LFP theta frequency is equal to the time taken for the fast wave to overtake the slow envelope, i.e. from Equation (A3.5):

$$f_\theta = \frac{v_p - v}{\lambda} \tag{A3.8}$$

so that the LFP theta frequency is simply the frequency of the interference pattern of the two components.

A4: Model of Coordinated Assembly Dynamics

While the above sections assumed that cells are are independent of each other once their mutual dependence on the animal's location and the LFP theta rhythm are accounted for, we now model the case in which cells have additional interdependencies arising from interactions within the local CA1 population. Therefore, we introduce a weight function through which the spiking activity of any given cell will influence the probability of another cell to spike:

$$w_{ij} = \frac{w_E}{\ell} e^{-(x_c^i - x_c^j)/\ell} \Theta\left(x_c^i - x_c^j\right) - w_I \tag{A4.1}$$

where w_E is the magnitude of the excitatory weights, ℓ is the peer interaction length, Θ is the Heaviside step function, w_I is the magnitude of the inhibitory weights and x_c^i, x_c^j are the place

field centers of cells i and j. This weight function is comprised of two components - an excitatory feedforward interaction with an interaction length ℓ, and a global inhibitory interaction.

To incorporate the dynamics of peer interactions into our existing single cell model, we follow a similar approach to that used by Harris et al. (2003) when estimating peer interactions from data. Specifically, peer spike trains are smoothed in time:

$$s_i(t) = \frac{1}{\sqrt{2\pi\tau^2}} \sum_{t_{ij}<t} e^{-(t_{ij}-t)^2/2\tau^2} \tag{A4.2}$$

where the smoothing kernel τ determines the peer interaction timescale - i.e., the timescale at which spiking activity from one cell will influence the activity of another cell (cf., the peer prediction timescale defined by Harris et al. (2003)). Here, s_i is the smoothed spike train of cell i and t_{ij} is the jth spike of cell i, where the sum is over j only. Note that the above sum is causal, i.e. only previous spikes will influence present and future activity. The influence of a set of peer spike trains on a particular cell i is then determined by the peer factor $P_i(t)$:

$$P_i(t) = g\left(\sum_j s_j(t) w_{ij}\right) \tag{A4.3}$$

which is the weighted sum of the smoothed peer spike trains, followed by a nonlinear transformation g:

$$g(x) = \begin{cases} x+1 & \text{if } x \geq 0 \\ \exp(x) & \text{if } x < 0 \end{cases} \tag{A4.4}$$

We note that, apart from some minor adjustments, Equations (A4.2-A4.4) are essentially an inversion of the peer prediction method of Harris et al. (2003) such that, with our particular choice of weights, we can generate data for an interacting population rather than estimate these interactions from that data.

The firing rate of each cell is simply modelled as the product of the activity generated by phase and rate coding alone with the additional peer factor $P_i(t)$. To simplify notation, we let $P_i(t) = P(x_c, t)$ where x_c is the place field centre of cell i. The total firing rate of the cell is then:

$$r(x_c, t) = A \exp\left(-\frac{(x_c - vt)^2}{2\sigma^2}\right) \exp\left(k \cos\left(\kappa x_c - \omega t + \psi_s\right)\right) P\left(x_c, t\right) \tag{A4.5}$$

A5: Analysis of Sigmoidal Phase Dynamics

Here we propose a model for the membrane potential (MPO) phase which matches the above traveling wave model for cells in the population that are spiking, but which differs outside the place field where the cell is silent. Since the above models were based on considerations of data from spiking neurons, the extension of a linear phase gradient to the MPO outside of the place field is not guaranteed, and several studies support a sigmoidal phase gradient (Chance, 2012; Diba and Buzsáki, 2008).

To model a sigmoidal phase gradient, we assume that the MPO frequency of a place cell depends only on the distance of the animal from the center of the place field x_c (Figure 8C):

$$f(x_c, t) = f_\theta + \Delta f \exp\left(\frac{-(x_c - vt)^2}{2\sigma^2}\right) \tag{A5.1}$$

where Δf is the increase in MPO frequency at the center of the place field. We can then calculate the phase of the MPO as a function of time, as the rat moves through the field at constant velocity v:

$$\begin{aligned}\psi(x_c, t) &= 2\pi \int_0^t f(x_c, \tau)d\tau + \psi(x_c, 0) \\ &= 2\pi f_\theta t - \frac{\pi^{3/2}\sqrt{2}\Delta f \sigma}{v}\left(\operatorname{erf}\left(\frac{x_c - vt}{\sqrt{2}\sigma}\right) - \operatorname{erf}\left(\frac{x_c}{\sqrt{2}\sigma}\right)\right) + \psi(x_c, 0) \end{aligned} \tag{A5.2}$$

If each cell precesses from 2π to 0, we can set $\lim_{t\to-\infty} [2\pi f_\theta t + \theta_s - \psi(x_c, t)] = 2\pi$ and $\lim_{t\to+\infty} [2\pi f_\theta t + \theta_s - \psi(x_c, t)] = 0$ to find $\psi(x_c, 0)$, so that:

$$\psi(x_c, t) = 2\pi f_\theta t - \pi \operatorname{erf}\left(\frac{x_c - vt}{\sqrt{2}\sigma}\right) + \theta_s - \pi \tag{A5.3}$$

which also gives $\Delta f = v/(\sqrt{2\pi}\sigma)$. This model reduces the number of parameters by explaining the rate of phase precession f_ϕ (Equation (A1.5)) purely in terms of the place field width σ, eliminating the second scale parameter R from the model and allowing experimental predictions based on fewer alterable parameters.

A6: Extension of Models to Two Dimensional Navigation

We now consider the population activity for arbitrary trajectories $\mathbf{x}(t)$ in two dimensions.

Linear Phase Gradient Model

For the linear phase gradient model, the most obvious extension is to modulate the direction of the traveling wave with heading direction, via a wave vector $\boldsymbol{\kappa}(t) = \kappa \mathbf{v}(t)/v(t)$. It is necessary to align the wavenumber with heading direction to account for data showing phase precession in two dimensions regardless of the direction of travel through a place field (Huxter et al., 2008). The previous results can then be extended using the phase:

$$\begin{aligned}\psi(\mathbf{x_c}, t) &= \int_0^t \omega(t')dt' - \boldsymbol{\kappa}(t) \cdot \mathbf{x_c} + \psi(\mathbf{x_c}, 0) \\ &= 2\pi f_\theta t + \frac{\Delta\phi}{2\pi}\frac{\pi}{R}\left(\int_0^t v(t')dt' - \frac{\mathbf{v}(t) \cdot \mathbf{x_c}}{v(t)}\right) + \psi_s \end{aligned} \tag{A6.1}$$

where we introduced an integral to account for variations in frequency due to changes in running speed along the trajectory and expanded out expressions for frequency and wavenumber. In this case, the phase offset $\psi(\mathbf{x_c}, 0)$ is the same for all neurons, since the phase gradient is enforced by the dot product term. In line with recent reports (Jeewajee et al., 2014), the relative firing phases of each cell depend on the current direction of motion rather than past trajectory in this model.

Sigmoidal Phase Gradient Model

For the sigmoidal phase gradient, the extension to navigation in two dimensions is straightforward. The frequency of the cell is simply a function of the distance to the center of the place field, and hence the phase is given by:

$$\psi(\mathbf{x_c}, t) = 2\pi f_\theta t + \frac{\sqrt{2\pi}}{\sigma} \int_0^t |\mathbf{v}(t')| \exp\left(-\frac{(\mathbf{x}(t') - \mathbf{x_c})^2}{2\sigma^2}\right) dt' + \psi(\mathbf{x_c}, 0) \qquad \text{(A6.2)}$$

where we used Equation (A5.1) and the expression for Δf.

A7: Remapping with Fixed Phase Sequences

In this section the structure present in a population of place cells with a linear phase code in two dimensions is described, not considering any rate coding. We then investigate the constraints when remapping between different place field configurations in an open environment, assuming the original set of phase lags between cells in the population is fixed. We then present a set of transformations which obey these constraints, allowing a prediction of the set of possible remappings in a network with a fixed set of theta phase lags.

Definition of a Phase Chart

In the linear traveling wave model, the MPO phase $\psi(\mathbf{x_c}, t)$ in each cell can be separated into a temporal component ωt and a spatial component $\theta_P = \boldsymbol{\kappa} \cdot \mathbf{x_c}$ which sets a fixed phase lag between any two cells in the population (see Equation (A6.1)). Since the temporal component is the same for each cell, we can disregard the temporal dynamics and focus only on the spatial component θ_P when analyzing the properties of networks with fixed phase lags.

During each theta cycle, a phase sequence is swept out. Only those cells within the rate coded area around the rat fire an action potential during a phase sequence, but every single place cell can be assigned an MPO phase, whether or not it fires. In the linear traveling wave model the relative phase between two cells $\Delta\theta_P$ depends only on the direction the rat is moving (determined by $\boldsymbol{\kappa}$) and the relative place field locations (determined by $\mathbf{x_c}$ for each cell).

If the rat is running in the direction $\boldsymbol{\kappa}$, the set of cells with a phase θ_P have place fields $\mathbf{x_c}$ given by:

$$C(\theta_P, \boldsymbol{\kappa}) = \left\{\mathbf{x_c} \in \mathbb{R}^2 | \ \mathbf{x_c} \cdot \boldsymbol{\kappa} \equiv \theta_P \bmod 2\pi\right\} \qquad \text{(A7.1)}$$

Equation (A7.1) shows that, in the linear traveling wave model, the set of cells with the same phase corresponds to periodic parallel lines extending across the whole environment (Figure A3). Each $C(\theta_P, \boldsymbol{\kappa})$ is a cell assembly active at phase θ_P in the sequence ordered along the direction $\boldsymbol{\kappa}$. A phase sequence consists of the set of cell assemblies $C(\theta_P, \boldsymbol{\kappa})$ with phases $\theta_P \in [0, 2\pi)$ for a particular $\boldsymbol{\kappa}$. In turn, a phase chart is the full set of phase sequences consisting of one $\boldsymbol{\kappa}$ for each running direction.

In this description, we have treated the set of cells as a two dimensional continuous sheet, so that we can assign a place field to each point in a two dimensional environment. This is an idealized case, but a finite sampling of cells from this idealized case would not affect any of the

arguments here. Moreover, since we are neglecting rate coding in this analysis, the definition of a phase sequence describes the sequence of MPO phases of all cells in the population, rather than the sequence of spikes which are localized to the vicinity of the animal.

Constraints Under Remapping with a Fixed Phase Chart

We now assume that this phase chart remains fixed under remapping, so that the set of phase lags remains, even though the cells can be assigned new place fields. This situation might arise if the phase differences between cells in the population is fixed anatomically, for example by upstream pacemakers. If $\mathbf{x_c}$ is the location of a place field before a remapping f, we denote the remapped place field as $\mathbf{x'_c} = f(\mathbf{x_c})$. Here, we set constraints on the possible remappings which ensure that the new place fields still display spatially ordered sequences under the same phase chart.

Constraint 1

After remapping, the new phase sequences should still sweep out paths in the environment, i.e. they should map out parallel lines which are ordered along a direction of movement, and each should represent a unique such direction as before.

For the remapped assemblies within a phase sequence to map out straight lines as in the original place field configuration, we require that:

$$f(C(\theta_P, \boldsymbol{\kappa})) = C(\theta'_P, \boldsymbol{\kappa}') = \left\{\mathbf{x'_c} \in \mathbb{R}^2 | \ \mathbf{x'_c} \cdot \boldsymbol{\kappa}' \equiv \theta'_P \bmod 2\pi\right\}, \tag{A7.2}$$

for some new direction $\boldsymbol{\kappa'}$.

Constraint 2

Since the phase lags are preserved, we require that:

$$\Delta\theta'_P = \Delta\theta_P \tag{A7.3}$$

for each pair of assemblies within each phase sequence.

In words, these constraints are 1) parallel lines of place fields are mapped to parallel lines and 2) phase differences among these parallel lines are preserved.

Affine Transformations Allow Remapping within a Fixed Phase Chart

Affine transformations have the property that sets of parallel lines remain parallel (constraint 1). They also preserve the ratios of distances along straight lines, meaning that constraint 2 is automatically satisfied, although with a possible spatial scaling.

We can demonstrate mathematically that the affine transformation:

$$\mathbf{x_c} \rightarrow M\mathbf{x_c} + \mathbf{a}, \quad M \in GL_2, \quad \mathbf{a} \in \mathbb{R}^2 \tag{A7.4}$$

satisfies the above constraints (GL_2 is the group of all invertible 2×2 matrices, called the general linear group), as shown below:

$$\text{Given} \quad \mathbf{x_c}\cdot\boldsymbol{\kappa} \equiv \theta_p \bmod 2\pi \quad \text{and} \quad \mathbf{x_c'} = \mathrm{M}\mathbf{x_c} + \mathbf{a} \tag{A7.5}$$

$$\text{Let} \quad \boldsymbol{\kappa'} = \left(M^{-1}\right)^T \boldsymbol{\kappa}, \quad \text{and} \quad \theta'_P = \theta_P + \mathbf{a}\cdot\left(\left(M^{-1}\right)^T \boldsymbol{\kappa}\right) \tag{A7.6}$$

$$\text{Then} \quad \mathbf{x_c'}\cdot\boldsymbol{\kappa'} = \mathbf{x_c}\cdot\boldsymbol{\kappa} + \mathbf{a}\cdot\left(\left(M^{-1}\right)^T \boldsymbol{\kappa}\right) \tag{A7.7}$$

$$\Longrightarrow \mathbf{x_c'}\cdot\boldsymbol{\kappa'} \equiv \theta'_P \bmod 2\pi, \tag{A7.8}$$

so that subsets with a given wave vector $\boldsymbol{\kappa}$ and phase θ_P are transformed to a new wave vector $\boldsymbol{\kappa'}$ and phase offset θ'_P. The transformation M can include scaling - in order to preserve phase precession, such a scaling would require a commensurate scaling of place field size in the direction of $\boldsymbol{\kappa'}$. Clearly, the transformation preserves phase differences along each direction.

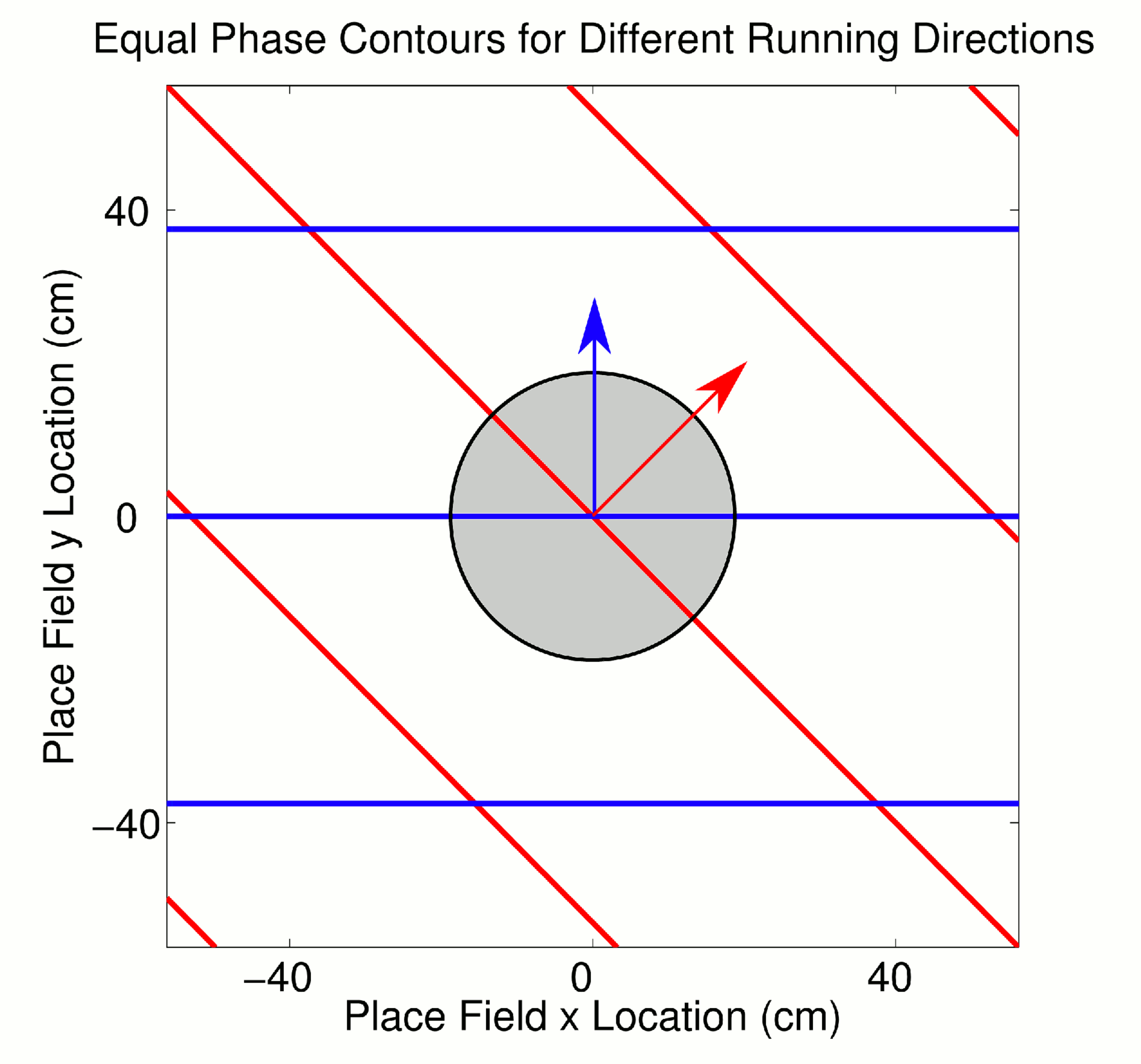

Figure A3: **Equal phase contours in the linear traveling wave model.**
The set of cells with a given phase θ_P maps onto parallel lines in the environment with spacing equal to the size of a place field and orientation aligned with the current running direction (shown by arrows). The phase lag between any two cells is fixed according to a linear population phase gradient $\boldsymbol{\kappa}$ along the direction of movement. The gray circle illustrates the spiking population, but the phase lags between cells are independent of this rate code.

References

Buzsáki, G. (2002). Theta Oscillations in the Hippocampus. *Neuron*, 33(3):325–340.

Chance, F. S. (2012). Hippocampal phase precession from dual input components. *J Neurosci*, 32(47):16693–703a.

Diba, K. and Buzsáki, G. (2008). Hippocampal network dynamics constrain the time lag between pyramidal cells across modified environments. *J Neurosci*, 28(50):13448–13456.

Geisler, C., Diba, K., Pastalkova, E., Mizuseki, K., Royer, S., and Buzsáki, G. (2010). Temporal delays among place cells determine the frequency of population theta oscillations in the hippocampus. *PNAS*, 107(17):7957–7962.

Harris, K. D., Csicsvari, J., Hirase, H., Dragoi, G., and Buzsáki, G. (2003). Organization of cell assemblies in the hippocampus. *Nature*, 424(6948):552–556.

Huxter, J., Burgess, N., and O'Keefe, J. (2003). Independent rate and temporal coding in hippocampal pyramidal cells. *Nature*, 425(6960):828–832.

Huxter, J. R., Senior, T. J., Allen, K., and Csicsvari, J. (2008). Theta phase-specific codes for two-dimensional position, trajectory and heading in the hippocampus. *Nature Neuroscience*, 11(5):587–94.

Jeewajee, A., Barry, C., Douchamps, V., Manson, D., Lever, C., and Burgess, N. (2014). Theta phase precession of grid and place cell firing in open environments. *Philosophical transactions of the Royal Society of London.*, 369(1635):20120532.

Schmidt, R., Diba, K., Leibold, C., Schmitz, D., Buzsáki, G., and Kempter, R. (2009). Single-trial phase precession in the hippocampus. *J Neurosci*, 29(42):13232–13241.
